# Sacral metastases in a patient with BRAF V600E+ papillary thyroid carcinoma

**DOI:** 10.1093/jscr/rjad585

**Published:** 2023-11-10

**Authors:** Rohith M Bhethanabotla, Suresh Mohan, Derrick Lin, Prerak Shah

**Affiliations:** Drexel University College of Medicine, Philadelphia, PA 19129, United States; Division of Otolaryngology – Head and Neck Surgery, Department of Surgery, Yale School of Medicine, New Haven, CT 06510, United States; Department of Otolaryngology – Head and Neck Surgery, Massachusetts Eye and Ear, Harvard Medical School, Boston, MA 02214, United States; Department of Otolaryngology – Head and Neck Surgery, Massachusetts Eye and Ear, Harvard Medical School, Boston, MA 02214, United States

**Keywords:** papillary thyroid carcinoma, distant, metastases, spine, screening

## Abstract

Lack of consensus exists on an algorithm to screen for synchronous distant metastases in patients presenting with papillary thyroid carcinoma (PTC). A 68-year-old male presented with a 3 cm supraclavicular neck mass. Computed tomography (CT) scan revealed a 1.3 cm left thyroid lobe nodule and 3 cm left level 3 and 4 lymphadenopathy. Ultrasound-guided fine needle aspiration was positive for PTC. Patient underwent total thyroidectomy and lymph node dissection with molecular testing confirming *BRAF* V600E+ PTC. Six weeks post-operatively, he developed left hip pain and numbness. Magnetic resonance imaging (MRI) revealed a large sacral mass and multiple bony lesions confirmed to be osseous metastases. Given the relatively rapid report of hip pain after surgery, metastases were likely synchronous at presentation and may have been detected with earlier suspicion. Further investigation is necessary to systematically stratify risk of synchronous distant metastases in patients with metastatic PTC.

## Introduction

Papillary thyroid carcinoma (PTC) comprises roughly 80% of thyroid malignancies [1]. Surgery remains the mainstay of primary treatment for intrathyroidal and locoregionally metastatic disease [2]. Approximately 5% of patients present with aggressive tumors that may lead to recurrence after surgery, distant metastasis (DM), and subsequent mortality within 5 years [3]. Therefore, a high degree of clinical suspicion for DM is necessary when proceeding with surgical management of PTC.

Identifying high-risk patients with distant disease at presentation requires a standardized algorithm which is not clearly defined. Currently, risk stratification has been outlined in American Thyroid Association (ATA) guidelines identifying histopathological parameters and molecular markers such as B-RAF proto-oncogene (*BRAF*) and Telomerase Reverse Transcriptase (*TERT*) as factors representing potentially more aggressive disease [4]. The status of many of these prognostic features are often not known until final pathology results. Distant synchronous metastases may thus go undetected for several weeks after surgery in an asymptomatic patient [5]. Herein, we present a case of a patient with PTC with early sacral metastases and review the evidence for the approach to optimal management.

## Case report

**Figure 1:**
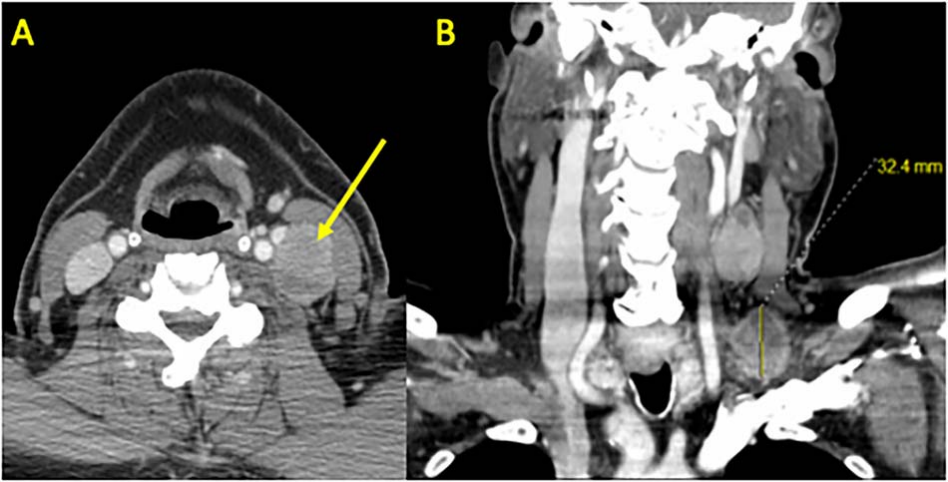
Preoperative CT Scan with Contrast. (**A**) Axial and (**B**) coronal sections demonstrating bulky left neck lymphadenopathy. Shown are a 3.2 × 2.8 cm level 4 mass in the supraclavicular fossa and a 3.9 × 2.3 cm level 3 mass adjacent to the internal jugular vein and carotid artery. A left thyroid 1.3 cm nodule was identified (not shown) and confirmed as PTC using fine needle aspiration.

**Figure 2:**
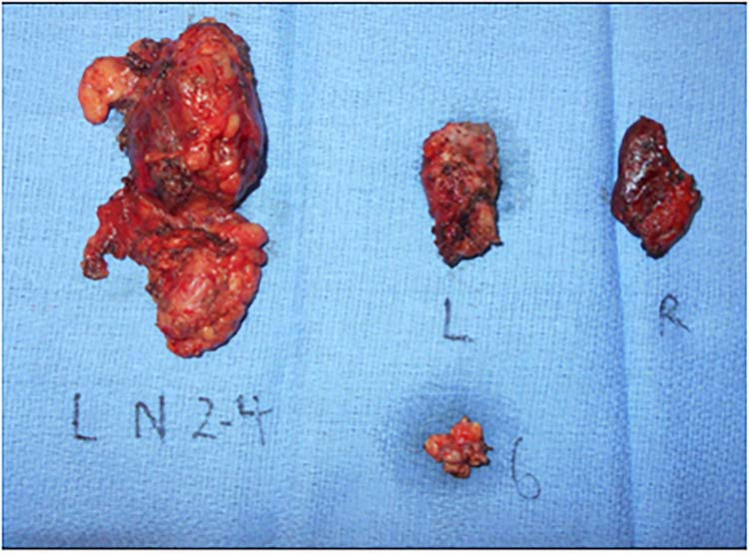
Gross pathology specimen of total thyroidectomy and neck dissection. Total thyroidectomy and left neck dissection specimen revealing a left thyroid 1.6 cm PTC and left neck level 2–4, 6 lymph nodes with final pathology yielding 16/42 positive nodes with extranodal extension and lymphovascular invasion. L, Left thyroid lobe; R, Right thyroid lobe, 6, level 6 lymph node packet; L N 2–4, left level 2–4 lymph node packet.

A 68-year-old male with prior history of non-Hodgkin’s lymphoma presented with 3-week history of progressively painful 3 cm supraclavicular neck mass. Computed tomography (CT) scan revealed a 1.3 cm left thyroid lobe nodule and 3 cm left level 3 and 4 lymphadenopathy (Fig. 1). Ultrasound-guided fine needle aspiration was positive for PTC. Two months later, the patient underwent a total thyroidectomy and central & left neck lymph node dissection (Fig. 2). Pathology showed a classical variant PTC, measuring 1.6 cm in the left lobe of the thyroid, with prominent fibrosis and lymphovascular invasion. Neck dissection of levels 2–4 and 6 yielded 16/42 positive nodes with presence of extranodal extension. Molecular testing revealed *BRAF* V600E positivity and staged as T1N1bMx.

**Figure 3:**
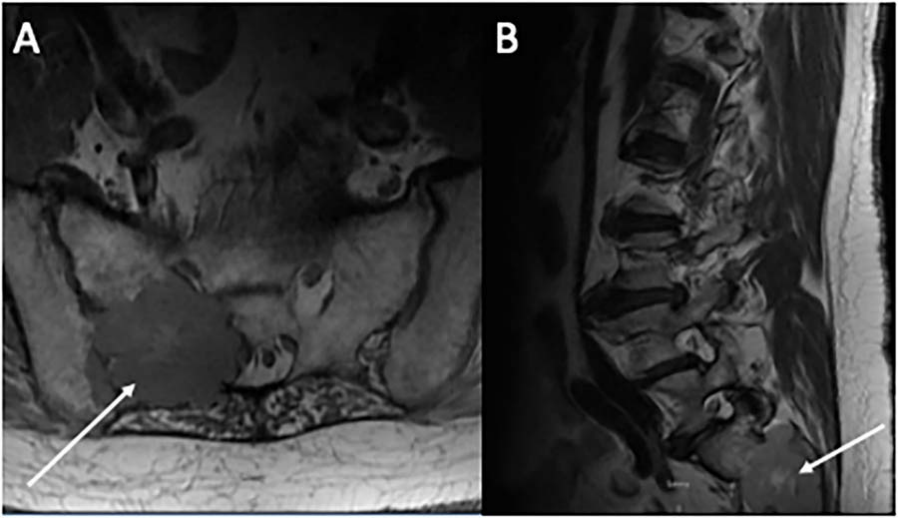
Magnetic resonance imaging of the lumbar spine. (**A**) Axial and (**B**) sagittal sections demonstrating an expansive 5 × 4 cm lesion in R sacroiliac joint. CT-guided core biopsy of the mass confirmed metastatic PTC.

**Figure 4:**
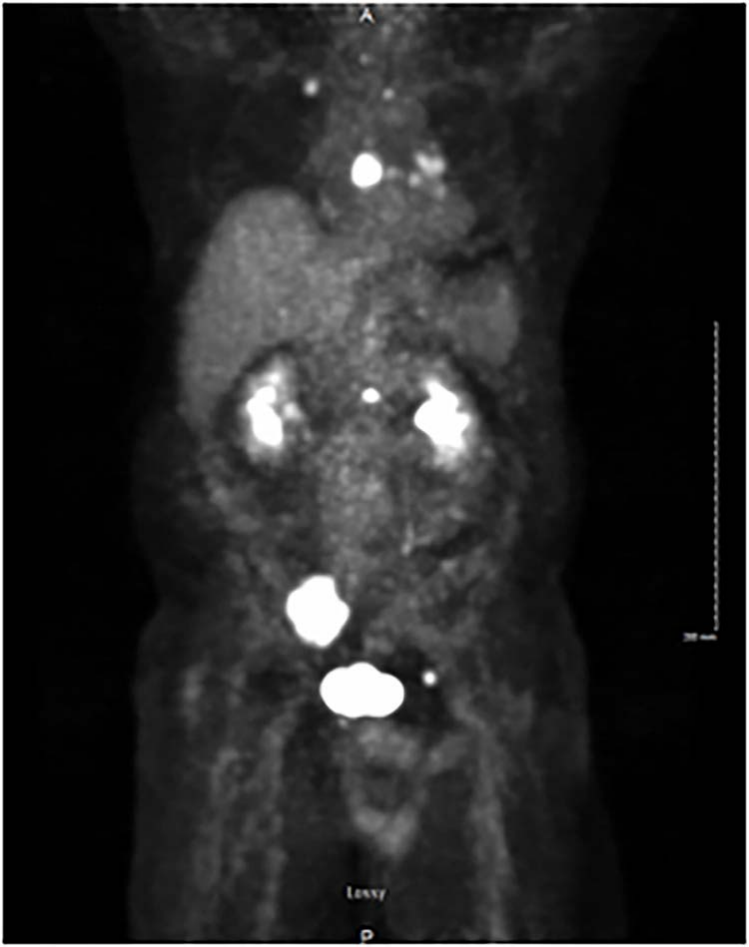
PET/CT demonstrating isotope uptake indicative of metastatic disease. Coronal section demonstrating no evidence of neoplastic disease in the head and neck region. Several foci of metastatic disease are present at the level of thoracic inlet. Shown are a 5 cm lytic metastatic focus involving R sacrum, 1.5 cm isotope positive focus of lytic metastatic disease involving the mid-portion of the sternum, and smaller positive foci involving left side of body of L-1, anterior aspect of left acetabulum, posterior aspect of 7th left rib, medial aspect of right clavicle and left transverse process of T-1.

A total of 6 weeks after surgery, while preparing for postoperative radioactive iodine therapy (RAI), the patient developed right hip pain and leg numbness. MRI of the lumbar spine revealed a 5.0 × 4.6 cm expansile lesion in the right sacroiliac joint (SI) and a herniated disk at L3-L4 (Fig. 3). CT-guided core biopsy of the mass confirmed metastatic PTC. He was treated with 165 mCi of I-131 and subsequent whole-body scan (WBS) showed uptake in the neck, right SI joint, and the posterior mediastinum. A positron emission tomography (PET-CT) scan demonstrated ^18^F-fluorodeoxyglucose (FDG)-avid mediastinal and left hilar adenopathy with no residual disease in the head and neck (Fig. 4). The patient was then treated with external beam radiation therapy with 40 Gy to the sacrum in 20 fractions. He was started on zoledronic acid, dabrafenib, and trametinib, but later switched to lenvatinib when follow up imaging showed persistent metastases. At 36 months following surgery, the patient remains on lenvatinib with PET-CT evidence of new peritoneal carcinomatosis and stable osseous metastases.

## Discussion

Herein, we report a case of locoregionally metastatic PTC with a large sacral lesion discovered 6 weeks post-operatively. While difficult to ascertain timing, the short onset is suggestive of synchronous DM that was unidentified at diagnosis. With most cancers, staging for DM occurs prior to definitive therapy. Thyroid cancer is unique in that staging is determined after resection of the primary cancer and in conjunction with imaging and thyroglobulin monitoring. Identification of DM early in the workup may facilitate timely and appropriate treatment to improve outcomes.

Historically, histopathological and clinical risk factors have been used to assess a patient’s risk of developing metastases. Various factors such as male sex, family history of thyroid cancer, advanced age >45 years, as well as tumor specific factors such as extrathyroidal extension, radioiodine resistance, lymph node metastases, and size of tumor larger than 2 cm portend poorer prognoses [6]. Many of these pathologic features used to prognosticate outcomes can only be confirmed after surgical intervention and therefore cannot be readily utilized in preoperative decision making.

The introduction of molecular testing with fine-needle aspiration biopsies has allowed for improved stratification of patients with thyroid cancer. In our patient, analysis determined positivity for *BRAF* V600E associated with aggressive phenotypic features in only 10–15% of cases [7, 8]. Some studies have shown *BRAF* associations with extrathyroidal extension, vascular invasion, multifocality, and lymph node metastasis leading to increased risk of recurrence and mortality. However, other studies have found minimally significant associations of *BRAF* mutation and independent variables such as age and sex [9–11]. Recently, interest has been given to *TERT* promoter mutations. When PTCs exhibit *BRAF* and *TERT* co-mutations, there is significantly increased risk for DM when compared to *BRAF-*only positive PTCs [12]. As we further investigate the pathophysiology of these cancers, identifying the genetic code of malignancies and stratifying subgroups at greater risk for DM will eventually lead to a paradigm shift in how we screen patients for metastatic disease.

Yip et al. developed a model of molecular-based risk stratification applicable to preoperative biopsy based on several late-hit driver mutations in PTC. They stratified patients into low-risk, intermediate-risk, and high-risk groups based on associations of molecular alterations and probability of disease aggressiveness (low risk included RAS and RAS-like variations, intermediate risk included BRAF V600E and other BRAF-like variations, and high-risk included early and late-hit *TERT* mutations). Using genetic profiles of different tumor types, they were able to establish risk of distant metastasis of 0.2–0.4% in low-risk profiles, 4.7–9.4% in intermediate-risk profiles and 19.3–33.5% in high-risk profiles [4]. As the correlation of the genetic mutation profiles on tumor characteristics and clinical behavior is better elucidated, these risk profile cohorts should demonstrate improved sensitivity and specificity in the subgroups at risk for metastatic disease.

Bone metastases are well-established in PTC. However, metastases in the absence of high-risk molecular features occur far less frequently. Identifying prognostic factors for DM at time of diagnosis to improve outcomes for patients with high-risk PTC remains an understudied area. Preoperative tumor marker identification and subsequent risk stratification can better inform more individualized targeted therapy strategies. With further research into optimal surveillance strategies, we anticipate marked improvement in long-term progression-free survival for patients with PTC and osseous metastases.

## Data Availability

The data that supports the findings of this study are available within the article.

## References

[1] Ramadan S , Ugas MA, Berwick RJ, Notay M, Cho H, Jerjes W, et al. Spinal metastasis in thyroid cancer. Head Neck Oncol 2012;4:39. 22730910 10.1186/1758-3284-4-39 PMC3466148

[2] Wexler JA . Approach to the thyroid cancer patient with bone metastases. J Clin Endocrinol Metab 2011;96:2296–307. 21816796 10.1210/jc.2010-1996

[3] Tuttle RM , Ball DW, Byrd D, Dilawari RA, Doherty GM, Duh QY, et al. Thyroid carcinoma. J Natl Compr Canc Netw 2010;8:1228–74. 21081783 10.6004/jnccn.2010.0093

[4] Yip L , Gooding WE, Nikitski A, Wald AI, Carty SE, Karslioglu-French E, et al. Risk assessment for distant metastasis in differentiated thyroid cancer using molecular profiling: a matched case-control study. Cancer 2021;127:1779–87. 33539547 10.1002/cncr.33421 PMC8113082

[5] Hindié E , Zanotti-Fregonara P, Keller I, Duron F, Devaux JY, Calzada-Nocaudie M, et al. Bone metastases of differentiated thyroid cancer: impact of early 131I-based detection on outcome. Endocr Relat Cancer 2007;14:799–807. 17914109 10.1677/ERC-07-0120

[6] Tufano RP , Teixeira GV, Bishop J, Carson KA, Xing M. BRAF mutation in papillary thyroid cancer and its value in tailoring initial treatment: a systematic review and meta-analysis. Medicine (Baltimore) 2012;91:274–86. 22932786 10.1097/MD.0b013e31826a9c71

[7] Xing M . BRAF mutation in thyroid cancer. Endocr Relat Cancer 2005;12:245–62. 15947100 10.1677/erc.1.0978

[8] Li X , Abdel-Mageed AB, Kandil E. BRAF mutation in papillary thyroid carcinoma. Int J Clin Exp Med 2012;5:310–5. 22993650 PMC3443896

[9] Vuong HG , Altibi AM, Duong UN, Ngo HT, Pham TQ, Tran HM, et al. Role of molecular markers to predict distant metastasis in papillary thyroid carcinoma: promising value of TERT promoter mutations and insignificant role of BRAF mutations—a meta-analysis. Tumour Biol 2017;39:1010428317713913. 29037127 10.1177/1010428317713913

[10] Moon S , Song YS, Kim YA, Lim JA, Cho SW, Moon JH, et al. Effects of coexistent BRAFV600E and TERT promoter mutations on poor clinical outcomes in papillary thyroid cancer: a meta-analysis. Thyroid 2017;27:651–60. 28181854 10.1089/thy.2016.0350

[11] Zhang Q , Liu S, Zhang Q, Guan Y, Chen Q, Zhu Q. Meta-analyses of association between BRAF(V600E) mutation and Clinicopathological features of papillary thyroid carcinoma. Cell Physiol Biochem 2016;38:763–76. 26871894 10.1159/000443032

[12] Xing M , Liu R, Liu X, Murugan AK, Zhu G, Zeiger MA, et al. BRAF V600E and TERT promoter mutations cooperatively identify the most aggressive papillary thyroid cancer with highest recurrence. J Clin Oncol 2014;32:2718–26. 25024077 10.1200/JCO.2014.55.5094 PMC4145183

